# A rare coexistence of papillary carcinoma and anaplastic carcinoma of thyroid in multinodular goitre: Case report and literature review

**DOI:** 10.1016/j.amsu.2020.06.024

**Published:** 2020-06-27

**Authors:** Sudha Shahi, Tika Ram Bhandari, Tridip Pantha, Dipendra Gautam

**Affiliations:** a Otorhinolaryngology Head and Neck Surgery, National Academy of Medical Sciences, Bir Hospital, Kathmandu, Nepal; b General Surgery, People's Dental College and Hospital, Kathmandu, Nepal

**Keywords:** Coexitent tumors, Mixed tumor, Papillary carcinoma, Multiodular goiter, Anaplastic thyroid carcinoma

## Abstract

**Introduction:**

Multinodular goiter is defined as multiple discrete nodules in the thyroid gland. The incidence of Papillary carcinoma thyroid was found to be highest out of total Multinodular Goiter cases while that of Anaplastic carcinoma was the least. We report a rare coexistence of Papillary carcinoma and Anaplastic carcinoma in adult patient with a long-standing Multinodular Goiter.

**Case presentation:**

Here we present a case of 54 years male with huge anterior neck swelling for 20 years with a gradual increase in size. Computerized tomography of neck revealed solidocystic mass lesion without any significant lymphadenopathy, features suggesting Multinodular goiter with differential diagnosis of Carcinoma Thyroid. Cytological examination showed Papillary thyroid Carcinoma. He underwent total thyroidectomy with central neck dissection. Postoperative period was uneventful. Histopathological report revealed features suggestive of mixed tumor of Papillary thyroid Carcinoma and Anaplastic Carcinoma thyroid TNM Staging T3 N0 M0, Stage IVA. After the final reports patient was sent for adjuvant therapy three weeks later where he received megavoltage external beam radiation and he was followed up till 12th week. He was assessed radiologically which showed no signs of physical progression of the disease. However, he was lost to follow up after that visit.

**Discussion:**

Long-standing benign conditions of thyroid can transform into malignant forms in the undefined duration of time.

**Conclusion:**

Regular follow up and early management of multinodular goiter at the right time can save a patient from undue stress and complication like the conversion into malignancy.

## 1 Introduction

Multi-nodular goiter (MNG) can be defined as an enlargement of the thyroid gland with palpable discrete nodules. It is more endemic in high mountainous areas like South-East Asia, Latin America and Central Africa. According to a report by World Health Organization in 2003, worldwide iodine deficiency rate was 9.8–56.9% while goiter prevalence was 4.7–37.3%.When speaking about malignancies in thyroid, Papillary thyroid carcinoma is the most common one.The incidence of Papillary carcinoma is also highest in MNG followed by Follicular carcinoma and anaplastic carcinoma [1]. Studies have also found the residual areas of papillary carcinoma in the setting of poorly differentiated or undifferentiated carcinomas. They are often termed as mixed tumor or collision tumors. Squamous metaplasia is one of the common events in such cases [2,3]. Similarly, coincidental ‘‘meeting’’ phenomenon or origin from a common stem cell have also been suggested [[4], [5]]. Concurrence of papillary carcinoma thyroid with medullary carcinoma, mucoepidermoid carcinoma, squamous carcinoma, liposarcoma, and anaplastic carcinoma follicular have been reported till date [[6], [7], [8], [9], [10]]. Among all the thyroid malignancies, anaplastic has the worst prognosis with a median survival between 3 and 9 months and 3-year overall survival rate of 10% [11,12]. Approximately 20–30% of patients are reported to develop anaplastic carcinoma from a pre or coexistent differentiated carcinoma following a multistep process of dedifferentiation which is mainly based on loss of the p53 oncogene suppressor. Molecular pathogenetic mechanisms of this transformation are still under study [[13], [14]]. In our case, the possible sequence of the disease might be the development of papillary carcinoma followed by dedifferentiation into anaplastic carcinoma. Though the surgical management is the preferred choice for both malignancies, anaplastic carcinoma is followed by adjuvant Radiochemotherapy [15]. This case has been reported according to SCARE criteria for case reports(16).

### 1.1 Case Presentation

**Fig. 1:**
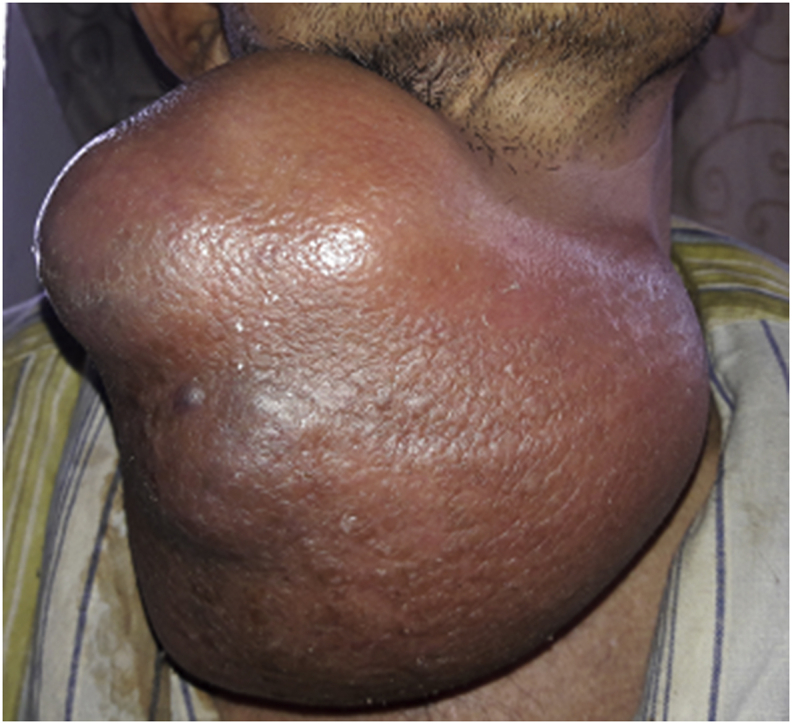
Figure showing Huge anterior neck mass.

**Fig. 2:**
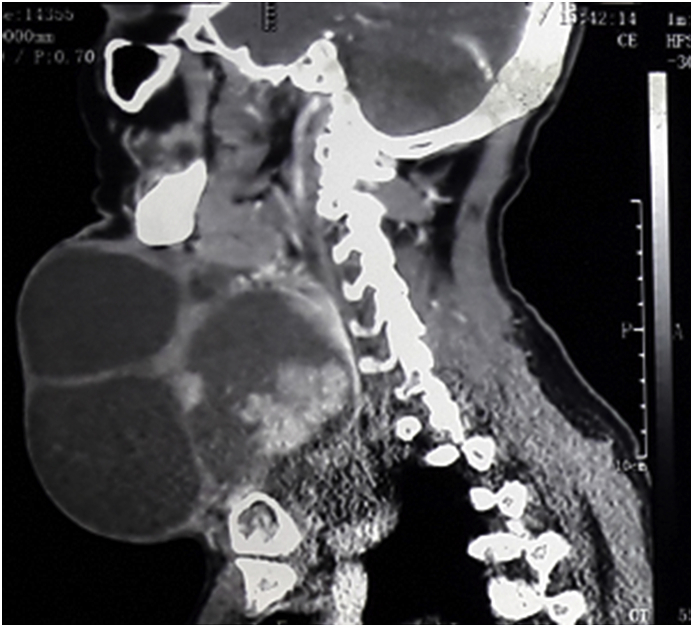
Figure showing plain Xray showing tracheal shift.

**Fig. 3:**
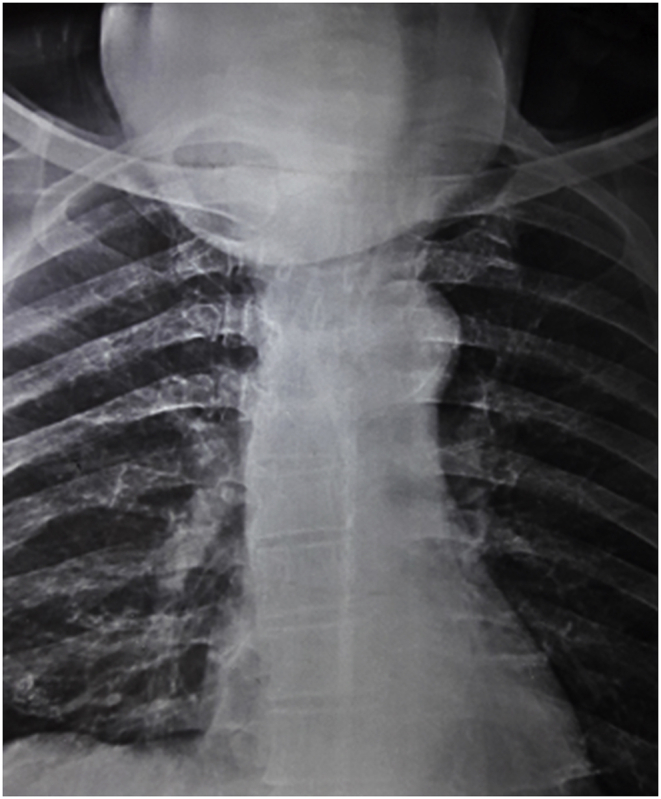
Figure showing CECT neck with multilocular solid cystic mass in neck.

**Fig. 4:**
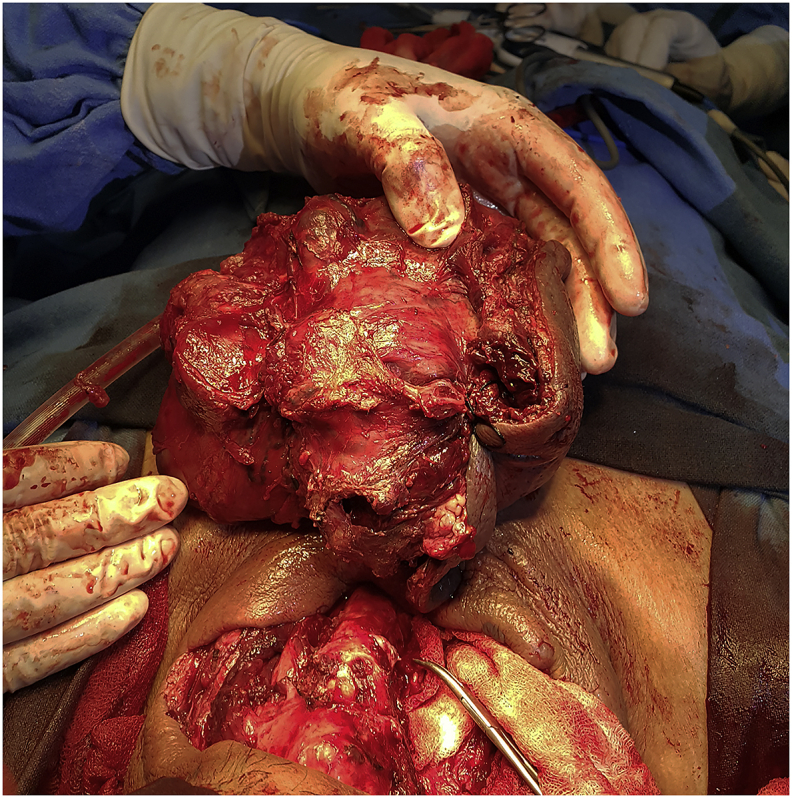
Figure showing Intraoperative thyroid mass.

**Fig. 5:**
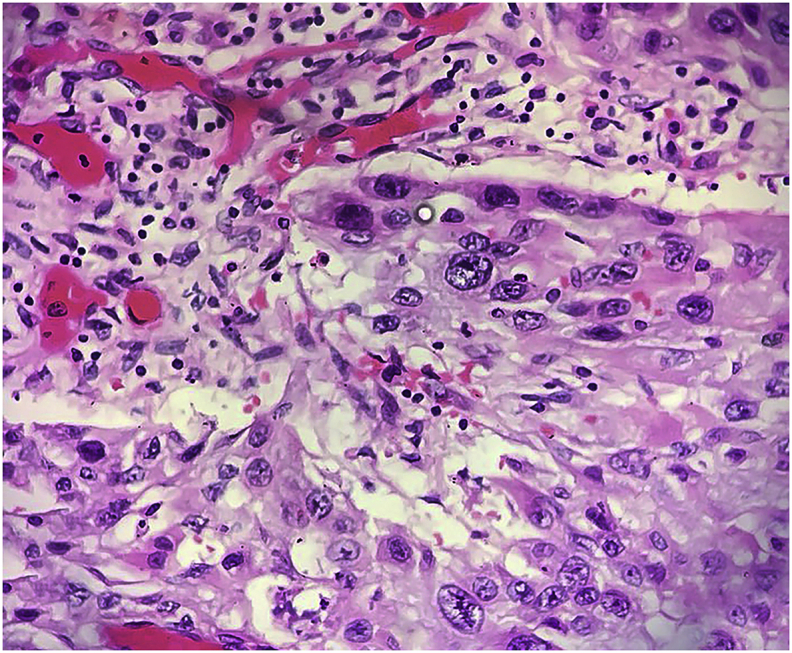
Figure showing Histopathological slide with mixed tumor.

A fifty-four years farmer presented with anterior neck swelling for 20 years (Fig. 1) The swelling was gradually progressive in nature. It was rapidly enlarging over the past 2 years large enough to disturb his daily activities like bending forward, lying supine etc. There was associated history of pain over the swelling for the past 6 months which was continuous, dull aching in nature.There was no history of dysphagia, odynophagia, shortness of breath initially but he started to develop dysphagia and shortness of breath on exertion over the last 6 months. Fever was present for last 1 week. There was a history of loss of appetite. Systemic examination revealed no abnormality. On examination of the swelling, there was a huge tender swelling measuring approximately 13 × 11 × 8cm^3^ over anterior neck extending from right posterior triangle to posterior border of left Sternocleidomastoid muscle. It was a tender, firm to hard multiloculated swelling with a well-defined margin and local rise in temperature. There was no fluctuation of the swelling, transillumination test was negative. There was no abnormal pulsation. There was an absence of thyroid bruit. Facial nerve function was intact on both sides. Ultrasonography (USG) of neck done two months back was suggestive of multinodular goiter. Similarly, the cytologic evaluation done two months back revealed Category II benign Colloid goiter. With the clinical history and findings above he was posed the diagnosis of Multinodular Goiter. He was then planned for contrast enhance computerized tomography of neck (CECT) (Fig. 2) which revealed multilocular predominantly cystic and matted solid mass lesion with its enhancement involving bilateral lobes predominantly in the right and isthmus pushing trachea towards left without any significant lymphadenopathy. Features were suggestive of multinodular goiter with differential diagnosis of carcinoma Thyroid. Repeat cytological examination was suggestive of papillary thyroid Carcinoma. His blood parameters were within normal range. Plain X-ray chest revealed a shifting of the trachea to the left (Fig. 3). Finally, with the diagnosis of papillary Carcinoma thyroid, he underwent total thyroidectomy with central neck dissection. Intraoperatively there was a huge multiloculated right thyroid mass measuring ~15 cm × 13 cm^2^ (Fig. 4) consisting of colloid material, adhered to surrounding structures. Few subcentimeter lymph nodes were identified at level VI. Both parathyroids and neurovascular structures were preserved. Postoperative period was uneventful with normal calcium levels (8.6 mg/dl). Histopathological report (Fig. 5) revealed features suggestive of mixed anaplastic carcinoma of the thyroid with papillary carcinoma; Pathological TNM grading pT3 N0 Mx: Stage IVa. After the final reports patient was sent for adjuvant therapy 3 weeks later where he received megavoltage external beam radiation delivered via intensity modulated radiation therapy (IMRT) and received 3Gy per dose fraction of radiotherapy with a total of 15 daily fractions. He was followed every 6 weekly post-treatment and on subsequent follow up on the 12th week he was assessed radiologically which showed no signs of physical progression of the disease. However, he was lost to follow up after the 4th visit.

## 2 Discussion

According to a study,the incidence of papillary carcinoma thyroid among total MNG cases was found to be 50%, Follicular was 30%, Lymphoma and Anaplastic Carcinoma was 10% each. Another group of tumors has been defined in literature known as mixed tumors where two distinctively different histopathological features are displayed in the same organ. The incidence of coexistence has been found to vary from 10% to 89% according to some studies [[1], [8]]. The first case of mixed type tumor was reported by CriIe in 1957 [17]. In a study done by Nel et.al, they found out of 82 patients with anaplastic carcinoma 5% were found to have anaplastic carcinoma with coexistent papillary carcinoma and 17% with coexistent follicular carcinoma [18]. Likewise, they found a coexistent tumor in 15% of those with a rapidly growing tumor and no previous thyroid disease, in 33% of those who presented with a slow-growing tumor, and in 27% of those who experienced accelerated growth in a preexisting thyroid swelling [6]. Several hypotheses have been suggested as mechanisms for collision tumors. Studies have suggested that the two primary tumors might occur by a chance or accidental ‘‘meeting.’’ Two different tumors may develop simultaneously if the region is altered by the similar carcinogenic stimuli. Another hypothesis suggests the presence of the first tumor alters the microenvironment leading to the development of the second adjacent tumor [19,20]. Similarly, recently accelerated growth of the long standing tumor has also been suggested as the cause for the coexistence of two or more types of thyroid carcinoma [21]. Our case was a mixed tumor with the coexistence of papillary ca thyroid and anaplastic carcinoma on longstanding Multinodular goiter. In our case though it was difficult to determine the sequence of events, if we look into the report by Crile in 1957, where he has suggested that stimulation of papillary or follicular carcinoma by thyroid-stimulating hormone for a prolonged duration of time could result into anaplastic carcinoma [17], we might agree that there was malignant transformation of multinodular goiter into papillary carcinoma thyroid followed by transformation into anaplastic carcinoma. Diagnosis of thyroid carcinoma mainly relies upon Physical Examination, USG, fine needle aspiration cytology (FNAC) and histopathological study for both types of carcinoma. Laboratory studies include CBC, Thyroid function test, liver function test, serum calcium, and phosphorus. Similarly, radiological studies like cross-sectional imaging of chest and neck that includes CT and MRI are helpful to determine the extent of disease for tumor staging and plan the most appropriate medical, surgical and or radiation therapy. The initial approach to management of patients with stage IVA (T1-T3a, N0/Nx, M0) or IVB ([T1-T3a, N1, M0] or [T3b, any N, M0] or [T4, any N, M0]) disease, depends on the resectability of a tumor where; T1: tumor <2cm size, T2: tumor >2 to < 4 cm, T3a: Tumor >4 cm limited to the thyroid, T3b: Gross extrathyroidal extension invading only strap muscles from a tumor of any size, Nx: Regional lymph nodes cannot be assessed, N0: No evidence of regional lymph nodes metastasis, and M0: No distant metastasis [22]. Two criteria are used to determine the tumor resectability for the curative purpose; (i) distinguishing between locoregional disease and distant metastatic disease, and (ii) extent of local invasion and the structures involved. Complete resection should be a goal in patients with stage IVa or Stage IVb since it is associated with good survival. Thus in stage IVa and IVb Anaplastic Thyroid Carcinoma, total thyroidectomy with a therapeutic central and lateral neck node dissection is recommended [15]. However, downstaging can also be considered for a locally unresectable tumor with high priority to airway preservation. Incidental Anaplastic Thyroid Carcinoma, on the other hand, is identified as a small, incidental finding after surgical resection of a non-anaplastic tumor. Complete resection is associated with a better prognosis. Role of adjuvant therapy is still questionable. However, recent data indicates apparent clinical benefit and possible improvement in survival from adjuvant-combined chemotherapy and radiation therapy in loco regionally confined Anaplastic Thyroid Carcinoma after complete/near complete resections or in those with the unresected disease. Still, treatment of intrathyroidal incidental Anaplastic Thyroid Carcinoma needs more discussions and more concrete grounds [23]. External beam radiotherapy has been found helpful in disease. However, trials involving combination chemotherapy and radiotherapy still need further evaluation for the treatment of anaplastic thyroid carcinoma [17]^.^ In our case, the scenario was a bit different where the preoperative diagnosis was made as Papillary carcinoma of thyroid based upon fine needle aspiration cytology (FNAC) and Radiological findings. Hence, total thyroidectomy with central neck dissection was performed. The diagnosis of Anaplastic Thyroid Carcinoma was added later after the final histopathological reports. Surgery was then followed by adjuvant radiotherapy 3 weeks later. On subsequent follow up every 6 weekly, there was no sign of disease progression.However, he was lost to follow up after his 4th visit.

## 3 Conclusion

Long-standing benign conditions of thyroid can transform into malignant forms in the undefined duration of time. The transition phase from the benign to the malignant condition is very subtle and difficult to trace. Thus regular follow up and management at the right time can save a patient from undue stress and complications. Early diagnosis and treatment of thyroid masses is important in all cases to prevent the conversion into malignancy.

## Ethical approval

As this is a case report, informed consent has been taken from the patient.

## Consent for publication

Written informed consent was obtained from the patient for publication of this case report and any accompanying images. A copy of the written consent is available for review by the Editor-in-Chief of this journal.

## Research registration unique identifying number (UIN)

Please enter the name of the registry and the unique identifying number of the study. You can register your research at http://www.researchregistry.com to obtain your UIN if you have not already registered your study. This is mandatory for human studies only.

## Guarantor

The Guarantor is the one or more people who accept full responsibility for the work and/or the conduct of the study, had access to the data, and controlled the decision to publish.

## Provenance and peer review

Not commissioned, externally peer reviewed.

## Funding

None.

## Declaration of competing interest

All authors declare that they have no competing interests.
